# Biosourced Disposable Trays Made of Brewer’s Spent Grain and Potato Starch

**DOI:** 10.3390/polym11050923

**Published:** 2019-05-26

**Authors:** Ana M. Ferreira, Jorge Martins, Luísa H. Carvalho, Fernão D. Magalhães

**Affiliations:** 1 LEPABE – Faculdade de Engenharia da Universidade do Porto, Rua Dr. Roberto Frias, 4200-465 Porto, Portugal; amf@fe.up.pt (A.M.F.); jmmartins@demad.estv.ipv.pt (J.M.); lhcarvalho@demad.estv.ipv.pt (L.H.C.); 2 DEMad- Departamento de Engenharia de Madeiras, Instituto Politécnico de Viseu, Campus Politécnico de Repeses, 3504-510 Viseu, Portugal

**Keywords:** starch, disposable trays, biosourced, brewers´ spent grain, beeswax, chitosan, gelatin, glyoxal

## Abstract

Single-use plastic items made of non-biodegradable and fossil-based materials have been identified as a major environmental problem in modern society. Food packaging materials represent an important fraction of these, and replacement with biosourced, sustainable and low-cost alternatives, is a key priority. In the present work, and for the first time, trays suitable for some food packaging applications were produced by the hot-pressing of brewer’s spent grains (BSG, a low added-value byproduct of the beer industry), bound with potato starch. Expanded polystyrene (EPS) trays were used as reference, since this material has been widely used in food packaging trays. The results demonstrated that all trays produced with varying proportions of BSG and potato starch have appropriate flexural strength, with values ranging between 1.51 ± 0.32 MPa, for 80% BSG content, and 2.62 ± 0.46 MPa, for 40% BSG content, which is higher than for EPS, 0.64 ± 0.50 MPa. Regardless of BSG content, flexural strength and modulus decreased significantly after contact with water, due to starch plasticization, attaining values below EPS. Trays produced with 60% BSG, and also with the addition of chitosan and glyoxal presented the highest flexural strength, both before and after contact with water, 3.75 ± 0.52 MPa and 0.44 ± 0.11 MPa, respectively. The latter is reasonably close to the reference value obtained for EPS.

## 1. Introduction

Europe produces about 25 million tons of plastic waste per year, with more than 60% resulting from packaging [1]. These petroleum-based plastics raise concerns about their disposal in the environment, due to the negative impact in the fauna and flora, with possible detrimental effects for human health. The development of low-cost, biodegradable packaging made from renewable resources is an alternative that has been receiving increasing attention. In the particular case of trays for food packaging, several studies have looked into biosourced alternatives, with emphasis on the combination of starch with different types of lignocellulosic fibers [2,3,4,5,6,7]. Production typically involves hot-pressing, with temperatures usually ranging between 130 to 220 °C, and the pressing times between 120 s to 20 min [2,3,4,6,7,8,9,10,11,12,13]. The different production parameters depend on the water content of the mixture, the pressing temperature, its density and the pressing apparatus.

Starch is a low cost raw material, renewable, biodegradable, and with good binding properties, especially on cellulosic substrates [14]. The usability of different forms of starch as raw material in a variety of applications, ranging from plastic utensils to wood particleboards, has been widely demonstrated [15,16,17].

Potato starch, in particular, is extracted from potatoes via a process that is relatively simple, when compared with extraction processes from cereals [18]. This type of starch presents a large granular size, high phosphorus content and low gelatinization temperature (61–68 °C) [18,19]. In 2017, the European Union produced 9.4 million tons of starch, of which 58% were for food, 2% for feed and 40% for non-food applications, mainly for paper-making companies [20].

Brewer’s spent grain (BSG) corresponds to the main by-product of the brewery industry, and comprises insoluble fragments of malt remaining after barley malt mashing and filtration [21,22]. Dried BSG is a lignocellulosic material containing about 192 to 284 g/kg of hemicelluloses (xylose and arabinose), 168 to 254 g/kg of cellulose (glucose), 153 to 247 g/kg of proteins, 119 to 278 g/kg of lignin, and 12 to 46 g/kg of ashes [23]. Approximately 3.4 Mt of brewers’ spent grain are produced every year in the European Union, and its main destiny is as animal feed [24]. Nonetheless, due to the high production volumes of BSG and the existence of other alternatives for animal feed, new applications for this low added-value by-product are desirable.

Beeswax is a natural wax produced by the younger working bees, and is used to build their honeycombs [25]. When secreted by the bees, the pure beeswax is almost white, but after contact with honey and pollen, its color turns yellowish [25]. Beeswax melts at temperatures between 63.5 °C and 64.5 °C, and is composed of linear wax monoesters and hydroxymonoesters (35–45%), complex wax esters (15–27%), hydrocarbons (12–16%), free fatty acids (12–14%), free fatty alcohols (approximately 1%) and residues of propolis, pollen and small pieces of floral components [25]. Beeswax has many applications, with the cosmetic and pharmaceutical industries being the main users [26]. Beeswax is also widely used as a waterproofing agent for wood and leather, and for strengthening threads [26].

Gelatin is a natural product obtained by the partial hydrolysis of collagen from animal by-products (bones, cartilages and skins) [27] and consists of proteins (85–96%), mineral salts and water [28]. Due to gelatin’s properties, it is widely used in the food, photographic and pharmaceutical industries [28].

Chitosan is a linear cationic polysaccharide obtained from crab and shrimp shells discarded by canning industries and fishing fleets [29], and has an enormous potential for different applications due to its properties.

This work studies, for the first time in the known literature, the combination of potato starch with BSG for the production of biodegradable trays. The mechanical properties are measured before and after water absorption. The effect of adding gelatin, chitosan, and a beeswax coating on water resistance is evaluated.

## 2. Materials and Methods

### 2.1. Materials

Brewer’s spent grain (23 wt % solids content), was kindly supplied by Super Bock Group, S.A. (Leça do Balio, Portugal). Potato Starch (81 wt % solids content) was obtained from Cargill (Schipol, The Netherlands). Glycerol USP was supplied by Consorima–Comércio de Produtos químicos, S.A. (Vila Nova de Gaia, Porto). Cotton pads (100% cotton) were bought in a local supermarket and distributed by Pingo Doce (Lisboa, Portugal). Beeswax was supplied by Mirosa, Fábrica de Ceras (Mira de Aire, Portugal). Glyoxal (40 wt %) was supplied by Sigma-Aldrich (Steinheim, Germany). Pure pork neutral collagen gelatin was bought in a local supermarket and distributed by A Colmeia do Minho (Aldeia de Paio Pires, Portugal). Chitosan (molecular weight around 300 kDa, degree of deacetylation > 85%) was supplied by Golden-Shell Pharmaceutical Co. Ltd. (Zhejiang, China). Expanded polystyrene (EPS) disposable trays were obtained from a local supermarket and presented a density of 32.7 ± 0.6 kg m^−3^, flexural strength, flexural strain and flexural modulus of 0.64 ± 0.05 MPa, 4.8% ± 0.8% and 0.28 ± 0.03 MPa, respectively, and a thickness of 0.56 ± 0.03 cm.

### 2.2. Methods

#### 2.2.1. Brewers’ Spent Grain Drying

Brewer’s spent grains (BSG), (23 wt % solids content) was dried in an oven at 105 °C for 7 h until it reached a solid content around 97%.

#### 2.2.2. Trays Production

Potato starch was mixed with previously dried BSG, glycerol (3.3 wt %) and deionized water in a 500 W hand mixer for 4 min. The proportions of the components in the different formulations are presented in Table 1. The solid content was 50% for all the formulations. Some formulations were also prepared with the incorporation of chitosan, gelatin and glyoxal (Table 1). The formulation with chitosan and glyoxal was prepared in two steps: In the first step, the potato starch, BSG, glycerol, glyoxal and water were mixed, and in the second step the chitosan solution (5 wt %) was incorporated right before the pressing stage, to avoid the formation of granules (due to reaction of chitosan with glyoxal at room temperature). The proportion of glyoxal towards gelatin and chitosan were based in the works of Spanneberg R. et al. [30] and Paiva D. et al. [15], respectively. The percentages of chitosan and gelatin in the formulations corresponded to the maximum percentage that did not considerably affect the expansion of the trays up to the top plate in the pressing stage.

After mixing the different components, single layer mats (54 g of mixture for all formulations) were hand formed in aluminum foil deformable containers (Lusoforma, Mem Martins, Portugal), with the following dimensions: 140 × 100 × 15 mm^3^. An in-house built parallel plate hot-press, manually controlled and equipped with thermocouples, was used to produce the trays. Metal frames with 5 mm (20% and 40% of BSG) and 4.3 mm (60% and 80% of BSG) thickness were used to impose the spacing between the press plates, and therefore define the final thickness of the trays. The trays with 60% and 80% of BSG required thinner metal frames (4.3 mm), as they did not expand as much as the ones with higher starch contents.

The hot-pressing process consisted of two stages. The first one comprised foaming of the mat to the desirable thickness, and the second one consisted in drying. The heated press is being used in place of a heated mold for shaping the starch/BSG mixtures. For the formulations with 20% and 40% of BSG, the first step was 20 s long, and for the formulations with 60% and 80% of BSG, it lasted 10 s to avoid the formations of clefts in the surface. The temperature of the press plates was 190 °C. The pressing started by placing the hand-formed mat in the press and lowering the top plate until it touched the metal frames. After the initial pressing time, the tray was removed from the press and the aluminum foil container cut out to facilitate drying.

**Figure 1:**
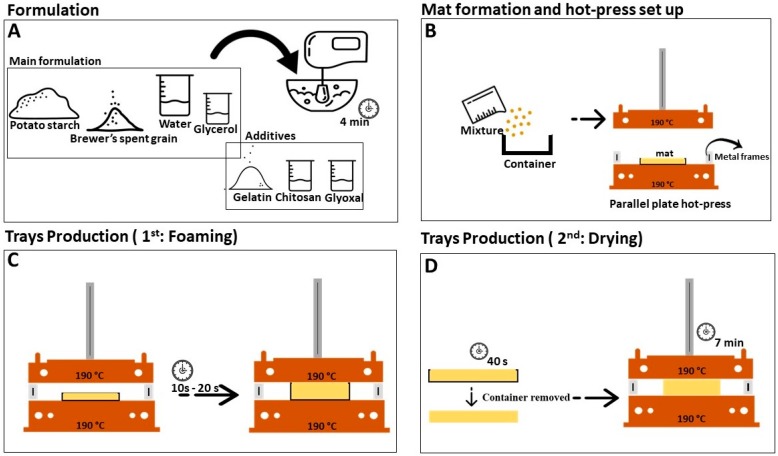
Schematic representation of the trays’ production steps.

This procedure lasted 40 s for all of the trays. Finally, the trays were placed again in the press, and the top plate lowered again until it was touching the metal frames. After 7 min the trays were removed and weighed. The final moisture content of the trays was between 47.8% and 46.0% after the first step and 1.3% and 3.0% after the second step. Strips with dimensions 100 mm × 25 mm were cut from the trays for testing. Figure 1 depicts the trays manufacture steps.

#### 2.2.3. Coating with Beeswax

Tray strips (100 mm × 25 mm) were coated by dipping for 15 s in beeswax (BW), which was previously melted in a water bath at 90 °C. The excess of BW was removed with a spatula. The final strips contained 24.6 ± 1.76 wt % of beeswax, since it not only coated the surfaces, but also penetrated into the porous structure. The coated trays were then dried at room temperature for 3 days.

#### 2.2.4. Trays Characterization

##### Scanning Electron Microscopic (SEM) Analysis

Scanning electron microscopic (SEM) analyses were performed with the Phenom XL microscope (Phenom-World B.V., Eindhoven, Netherlands) and the Secondary Electron Detector (SED). Tray pieces were mounted on stubs using carbon double-sided tape, and the surfaces were coated with a thin gold layer to increase the sample’s conductivity.

##### Density and Moisture Content

The density (kg/m^3^) was determined immediately before mechanical testing and calculated as the ratio of the weight and volume of the tray strips (100 mm × 25 mm). For each parameter, 4 to 7 tray strips were analyzed. The moisture content was determined by weighing the tray before and after the pressing steps.

##### Mechanical Properties

Flexural tests were performed using a Mecmesin MultiTest-1-d testing machine equipped with a Mecmesin BFG 200 dynamometer (Slinfold, United Kingdom). Before testing, the trays were conditioned in an environmental chamber at 20 °C and 60% relative humidity for 7 days, and then cut into strips (100 mm × 25 mm). The flexural tests were performed using the three-point bending rig and a span setting of 7.6 cm, according to EN ISO 178:2003 [31]. The strips were deformed until breaking, and the flexural strength (σ_f_), flexural strain (ε_f_) and flexural modulus (S_i_) were calculated.

**Figure 2:**
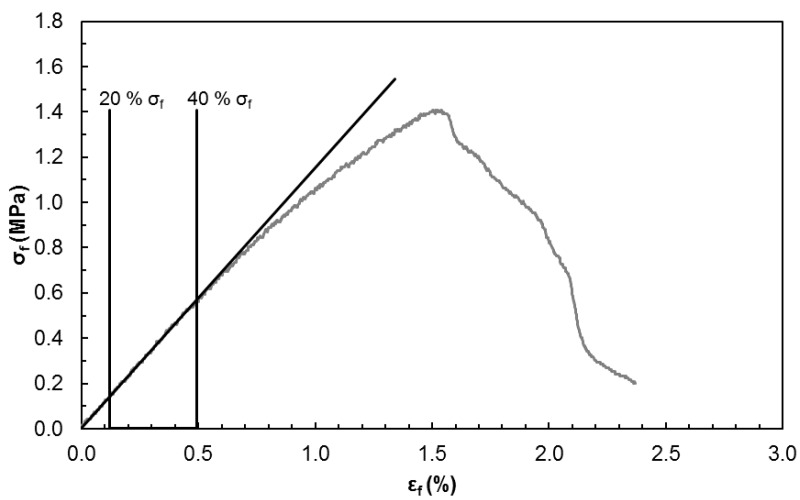
Stress-strain curve and corresponding slope determined between 20% and 40% of the maximum flexural strength.

The flexural modulus was defined as the slope of the stress-strain curve between 20% and 40% of the maximum flexural strength, to assure that all the values corresponded to the slope of the initial straight line portion. In Figure 2 is exemplified how this flexural modulus was determined. The crosshead speed was 2.0 mm/min. Data reported are an average of 4 to 7 replicates.

##### Water Resistance

Rectangular cotton pads (15 mm × 25 mm) soaked in water (90 wt %) were placed on top of the center of the tray strips (100 mm × 25 mm), and the system was placed in a closed container for 24 h at room temperature. The percentage of the water absorbed, relative to the sample’s initial weight, was determined by weighing the tray strips before and after the essay. Flexural tests were also performed after the test to evaluate the mechanical properties of the trays after being in contact with water. Data reported are an average of 4 to 7 replicates.

## 3. Results and Discussion

**Figure 3:**
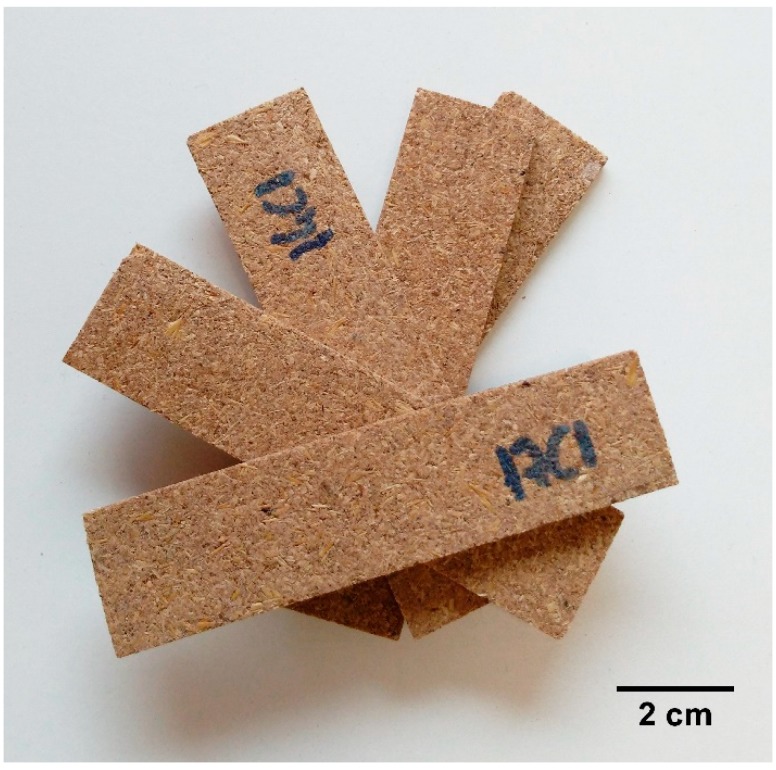
Samples (10 cm × 2.5 cm × 0.43 cm) cut from trays produced with 60% BSG.

In the present work, different compositions were tested for the production of disposable biosourced trays using brewer’s spent grain (BSG) and potato starch as main raw materials. Firstly, the impact of BSG content on the mechanical and water resistance properties of BSG/starch trays was studied. Then, four additives (gelatin, chitosan, glyoxal and beeswax) were tested in order to evaluate their effectiveness in improving water resistance. As an example, Figure 3 shows samples cut from trays with 60% BSG, used for testing.

### 3.1. Brewer’s Spent Grain Content

**Figure 4:**
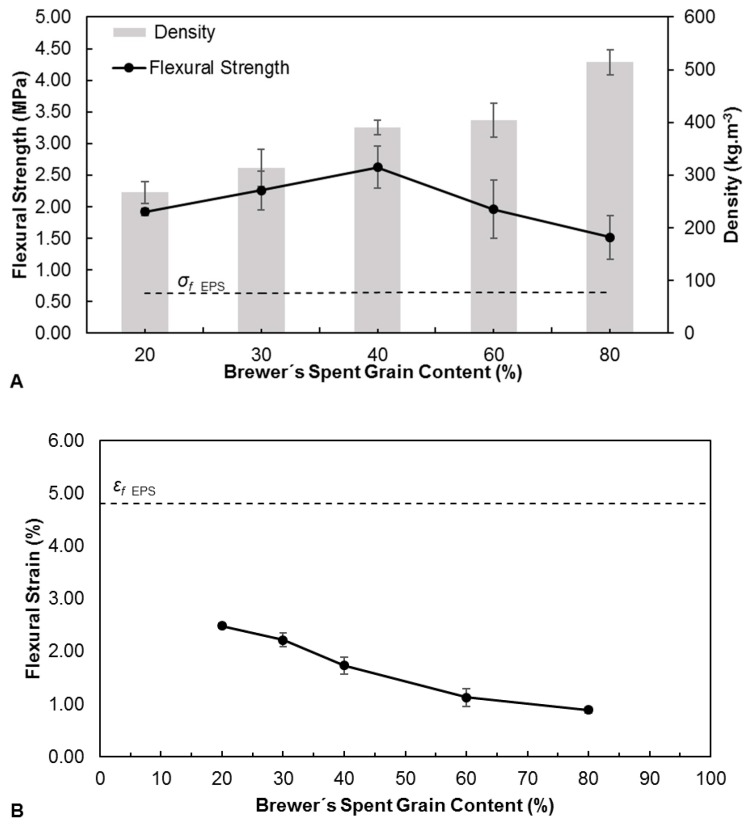

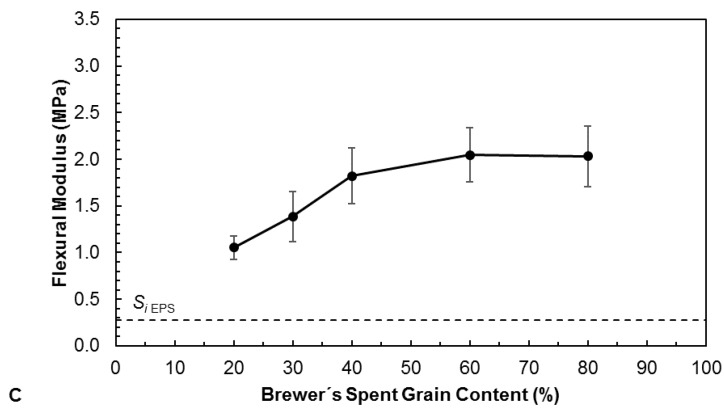
Flexural strength and density (**A**), flexural strain at break (**B**) and flexural modulus (**C**) of trays produced with different contents of Brewer’s Spent Grain, defined as the weight fraction in the BSG/starch dry mixture. The horizontal dashed line represents the flexural strength of expanded polystyrene (EPS) trays.

Figure 4 (Graphic A) presents the flexural strength of the trays produced with different BSG contents and their respective densities. The density tends to increase with BSG content. This is expected, since gelatinized starch is able to retain water vapor bubbles, resulting in a rigid foam as it dries [5]. This foam fills interparticular spaces, reducing the apparent density of the material. Decreasing starch content therefore results in higher densities, as less foam is formed. The flexural strength increases slightly up to a BSG content of 40%, and decreases afterwards. The initial increase may be mostly related to the increase in density, since this implies higher internal cohesion. However, above 40% BSG content, the strength starts to decrease, probably because the amount of starch is not sufficient to provide adequate interparticular bonding. All of the trays produced, regardless of BSG content, have flexural strengths (between 1.51 ± 0.32 MPa and 2.62 ± 0.46 MPa), that are higher than EPS 0.64 ± 0.50 MPa. It must be noted that trays with a 90% BSG content were also produced, but these were very fragile, breaking easily upon handling, and therefore were not further characterized.

Figure 4 (Graphic B) shows that all the trays produced with BSG have lower flexural strain at break (between 0.89% ± 0.05%) and 2.49% ± 0.05% than EPS 4.81% ± 0.84%. The flexural strain decreases as BSG content increases, showing that starch plays a role on the material’s flexibility. All the trays produced with BSG present higher flexural modulus (between 1.1 ± 0.1 MPa and 2.0 ± 0.3 MPa) than EPS (0.28 ± 0.03 MPa), in consonance with the lower flexural strain at break. The flexural modulus increases with BSG content up to 60%, and then tends to stabilize. The higher rigidity of the BSG/starch trays, in relation to EPS, is not necessarily a limitation. Besides, flexibility can be improved by increasing glycerol content, since it acts as a plasticizer for starch, even though this was not tested in this work.

**Figure 5:**
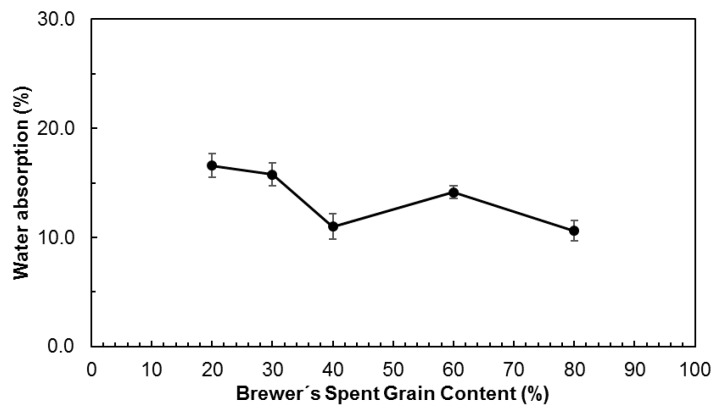
Water absorption, relative to the sample’s initial weight, of trays produced with different contents of Brewer’s Spent Grain.

Figure 5 presents the water absorption of the different trays after being in contact with a cotton strip with 90 wt % water content for 24 h. There is a slight tendency for water absorption to decrease as BSG content increases, from 16.6% ± 1.09% for 20% BSG to 10.6% ± 0.83% for 80% BSG content. This may be related to starch being more hydrophilic than BSG particles, and/or to hindered water penetration in the denser trays. For EPS water absorption was practically zero.

**Figure 6:**
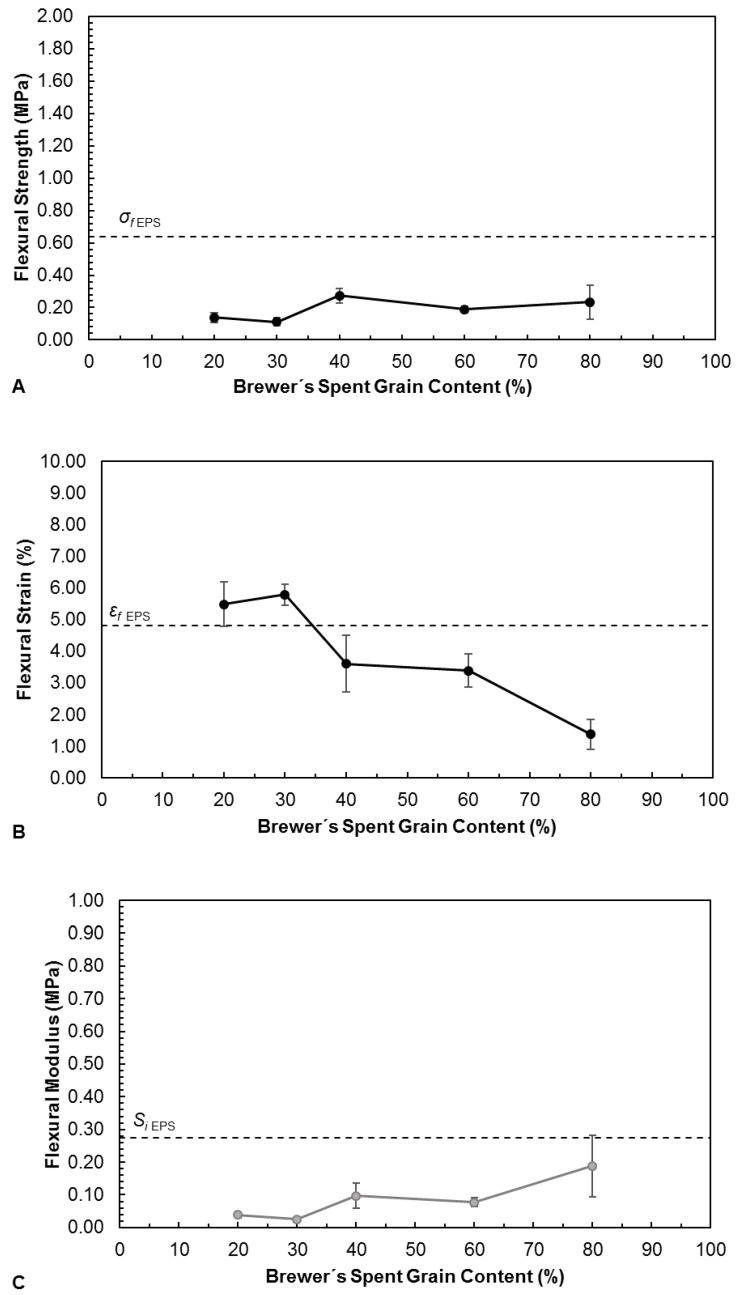
Flexural strength (**A**), Flexural strain (**B**) and Flexural modulus (**C**) at break of trays produced with different contents of Brewer’s Spent Grain, after water absorption. The horizontal dashed line represents the flexural strength of EPS trays.

Figure 6 presents the flexural strength, flexural strain at break, and flexural modulus of the trays, after the water absorption procedure. As expected, the flexural strength and flexural modulus, regardless of BSG content, decrease drastically, assuming values below EPS. This strong water sensitivity is typical of starch-based materials [5]. Flexural strength at break increases with water absorption, reaching EPS’s values for the two lowest BSG contents, which is also expected due to water’s plasticizing effect.

### 3.2. Trays Morphology

**Figure 7:**
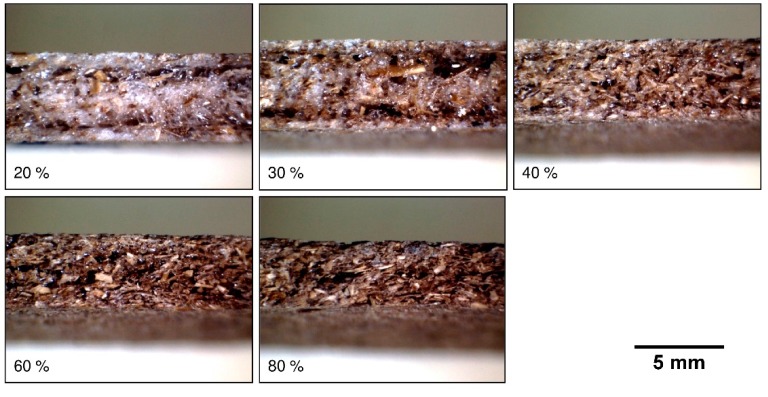
Macrographs (8× magnification) for cross-sections of the trays with different BSG contents (20%, 30%, 40%, 60% and 80%).

Figure 7 presents the tray’s morphology for different BSG contents. Interparticular starch foam is clearly visible in the tray with 20% BSG. The foam becomes progressively less visible as starch content decreases. The self-foaming capabilities of starches are well known, and have been used to produce low-density composite materials [16].

**Figure 8:**
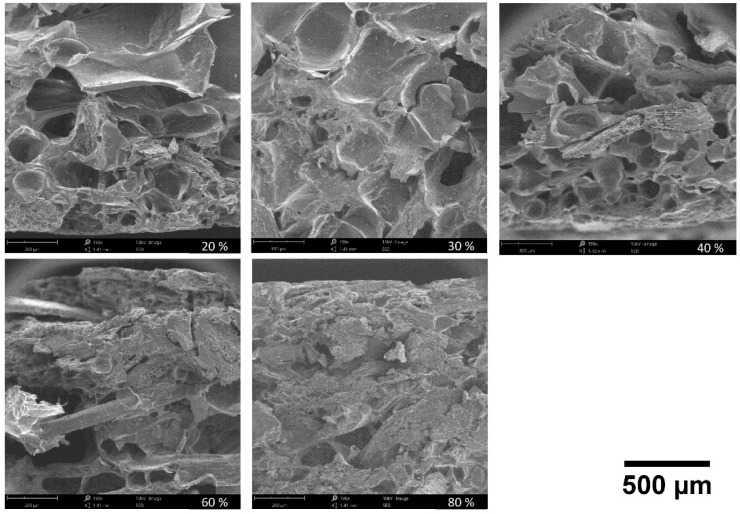
Scanning electron micrographs (190× magnification) for cross-sections of the trays with different BSG contents (20%, 30%, 40%, 60% and 80%).

Figure 8 presents SEM images of the different trays’ samples. The trays with lower BSG contents (20%, 30%, 40%) display starch foam cells with walls well adhered to BSG particles. For higher BSG contents a much denser morphology is observed, with less visible foam cells. These images evidence that water penetration may be easier in the trays with higher starch content, due to higher porosity.

### 3.3. Effect of Gelatin, Chitosan, Glyoxal and Beeswax on the Properties of BSG/Starch Trays

Based on the results obtained, the formulation with 60% BSG content was selected for further studies, due to its good mechanical properties. The purpose was to attempt decreasing water sensitivity by adding biosourced additives. Gelatin and chitosan were selected, as they are obtained from food by-products, and possess amine groups that are able to create electrostatic interactions with the phosphate groups of potato starch [5], and are chemically crosslinkable with glyoxal, a synthetic dialdehyde that can be obtained from natural sources. Beeswax was used as a natural hydrophobic coating.

**Figure 9:**
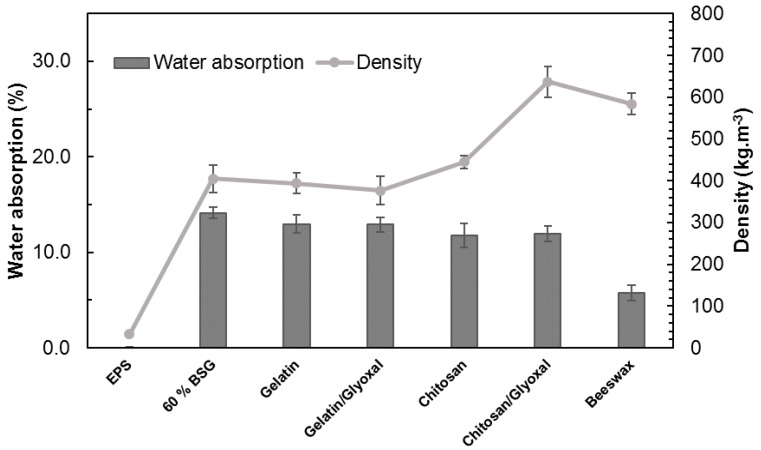
Weight variation of EPS samples and the trays with 60% of BSG and different additives (gelatin, gelatin and glyoxal, chitosan, chitosan and glyoxal and beeswax) after being exposed to a cotton strip (25 mm × 15 mm) with 90 wt % of moisture for 24 h. The respective densities are presented.

**Figure 10:**
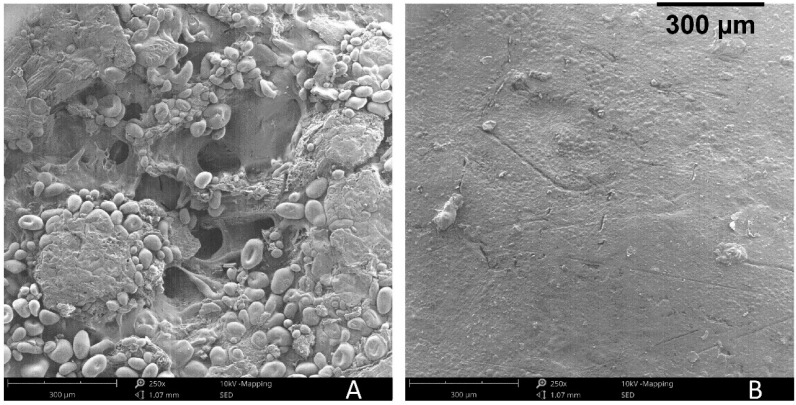
Scanning electron micrographs of the trays surfaces with 60% of BSG without (**A**) and with (**B**) beeswax coating.

Figure 9 presents the water absorption and density for the different trays. Water absorption decreased 8.4% with the addition of gelatin, and 17.0% with addition of chitosan, in comparison with the trays without additives. Use of glyoxal crosslinker did not improve the results. A more significant decrease in water absorption (59.6%) was obtained with the beeswax coating. Figure 10b shows how the beeswax coating covers the surface, limiting water penetration. The uncoated tray, seen in Figure 10a, displays an open structure, where BSG particles and foam cells are visible. Interestingly, ungelatinized starch granules are also visible in the surface. These are not present in the images of the trays’ cross sections. Probably, the starch granules in contact with the press platen dry out rapidly, as water vapor migrates from the surface towards the center, are therefore is unable to undergo the gelatinization process.

The trays with chitosan and chitosan/glyoxal presented high densities (445 ± 15.6 and 637 ± 37.2 kg m^−3^, respectively), because chitosan increases the viscosity of the liquid medium and impairs foam expansion. Presence of glyoxal accentuates this effect, since the crosslinker starts to react with chitosan’s amine groups even at moderate temperature, further increasing viscosity. The trays with beeswax were also denser than the BSG/starch reference (584 ± 16.4 kg m^−3^), due to the presence of the coating.

**Figure 11:**
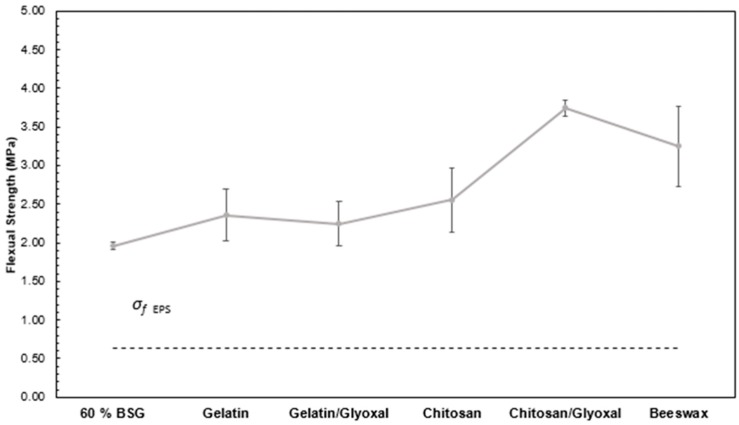
Flexural strength of trays with 60% of BSG and different additives (gelatin, gelatin and glyoxal, chitosan, chitosan and glyoxal and beeswax).

Figure 11 presents the flexural strength of the new trays. Addition of gelatin and chitosan yields slightly higher strength, but the highest values are obtained for the chitosan/glyoxal combination (3.75 ± 0.52 MPa), mainly due to the higher density, even though the occurrence of crosslinking may also play a role. It must be noted, however, that these trays were more difficult to produce and had more irregular surfaces, due to the higher viscosity of the mixture. Coating with beeswax also yields higher strength than the BSG/starch reference (3.25 ± 0.28 MPa), due to the improved cohesion within the surface layers provided by the coating.

**Figure 12:**
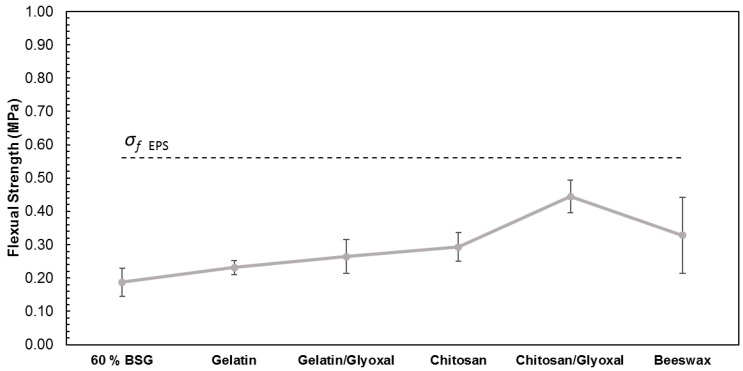
Flexural strength trays with 60% of BSG and different additives (gelatin, gelatin and glyoxal, chitosan, chitosan and glyoxal and beeswax) after waster absorption.

Despite the improved dry flexural strength, the negative effect of water absorption is still substantial, as seen in Figure 12. Nonetheless, the trays with chitosan/glyoxal yielded 0.44 ± 0.11 MPa and the ones with beeswax 0.33 ± 0.15 MPa. These values are much closer to the flexural strength obtained for EPS (0.56 ± 0.04 MPa). The large scattering seen for the beeswax-coated samples may be due to small defects in some coatings, allowing water penetration.

## 4. Conclusions

A serious problem is currently posed by plastics escaping recycling systems and ending up in landfills or being released into the environment, damaging terrestrial and marine ecosystems. The present work explores the viability of using low-cost biosourced and abundant raw-materials (brewer’s spent grain—a byproduct of the beer industry—and potato starch) to produce trays suitable for packaging. The results demonstrate that the natural composites produced with different BSG contents have higher flexural strength and rigidity than the EPS reference. After absorbing water, however, the flexural strength and flexural modulus decreased drastically, regardless of the BSG content, becoming lower than for EPS.

Combination of the starch binder with chitosan and glyoxal crosslinker, or coating the trays with beeswax, contributed to improve flexural strength after water absorption, allowing us to attain values closer to the EPS reference. The observed difficulty in processing the paste containing chitosan/glyoxal, due to the higher viscosity, indicates that further optimization of this formulation is needed in order to improve processability, while insuring an appropriate performance of the final product.

These results point out how a low-cost natural composite based on BSG bound with starch, which is easily processable by hot-pressing, is suitable for some packaging applications where EPS trays are traditionally used.

## Figures and Tables

**Table 1:** Compositions of the different formulations used to prepare the trays. BSG: Brewer’s Spent Grains.

BSG (g/100 g Solids)	Potato Starch (g/100 g Solids)	Glycerol (g/100 g Solids)	Deionized Water (g/100 g Formulation)	Gelatin (g/100 g Solids)	Chitosan (g/100 g Solids)	Glyoxal (g/100 g Solids)
20.0	76.7	3.3	50	-	-	-
40.0	56.7	3.3	50	-	-	-
60.0	36.7	3.3	50	-	-	-
80.0	16.7	3.3	50	-	-	-
60.0	35.7	3.3	50	1	-	-
60.0	35.7	3.3	50	1	-	0.02
60.9	32.7	3.3	49.6	-	3.0	-
60.0	32.1	3.3	50	-	3.0	1.6
